# Aquaporin 5 expression is altered in ovarian tumors and ascites-derived ovarian tumor cells in the chicken model of ovarian tumor

**DOI:** 10.1186/s13048-014-0099-x

**Published:** 2014-10-25

**Authors:** Anupama Tiwari, Jill A Hadley, Ramesh Ramachandran

**Affiliations:** Department of Animal Science, Center for Reproductive Biology and Health, The Pennsylvania State University, University Park, PA 16801 USA

**Keywords:** Water channel transmembrane protein, Malignancy, Metastasis, Invasiveness, Epithelial ovarian tumor

## Abstract

**Background:**

Aquaporin 5 (AQP5), a member of the aquaporin family of transmembrane channel proteins, is involved in water transport and cellular proliferation in various tumors. The objective of this study was to determine cellular localization of aquaporin 5 (AQP5) in the ovarian tumors of chicken, a preclinical model for human ovarian tumor and to determine if AQP5 mRNA and protein expression levels in cancerous chicken ovaries and in ascites-derived chicken ovarian cancer (COVCAR) cell lines are different from normal ovaries and normal ovarian surface epithelial (NOSE) cells, respectively.

**Methods:**

Immunohistochemical staining was performed to determine the localization of AQP5-immunoreactive (ir) cells in normal and cancerous ovaries. To determine AQP5 mRNA and protein concentrations in cancerous ovaries and COVCAR cell lines, quantitative real time PCR and Western blotting analysis were performed, respectively. Student’s t-test was performed to compare the levels of AQP5 mRNA or protein in cancerous ovaries and COVCAR cell lines with that of normal ovaries and NOSE cells, respectively.

**Results:**

AQP5-ir cells were localized in granulosa and theca layers of normal ovarian follicles whereas cancerous ovaries showed AQP5 immunostaining in the surface epithelium, fibroblast cells of the stroma, and in the cells lining tumor cysts and acini. AQP5 mRNA concentration were significantly lesser while AQP5 protein concentrations were significantly greater in cancerous ovaries compared to that in normal ovaries (P < 0.05). Whereas AQP5 mRNA concentrations were significantly greater while AQP5 protein concentrations were lesser (P < 0.05) in COVCAR cell lines compared with that in NOSE cells.

**Conclusion:**

AQP5 is differentially expressed in ovarian tumor and in COVCAR cell lines suggesting a potential involvement of AQP5 in ovarian tumorigenesis, metastasis, and survival of ovarian tumor cells in ascites.

## Introduction

Ovarian cancer is a very complex and heterogeneous disease that ranks fifth in cancer-related deaths among women [1,2]. High mortality due to ovarian cancer is attributed to diagnosis of the disease at advanced stages and high recurrence following first line of treatment. Advanced stages of ovarian cancers are usually associated with ascites formation [3]. Ascites is accumulation of fluid in the peritoneal cavity as a result of a combination of several factors including peritoneal lymphatic obstruction by tumor cells causing impaired lymphatic drainage [4], rupture of organ/tissue architecture leading to plasma leak into the peritoneal cavity [5], and secretion of vascular permeability factors by tumor cells [6]. Furthermore, imbalances in water transport could result from alterations in expression of water channel proteins such as aquaporin (AQP).

The aquaporins are a family of integral membrane proteins that facilitate water transport across the cell membrane [7]. In addition to water transport, aquaporins are reported to play a major role in angiogenesis, cell migration [8], cell proliferation [9], cell volume regulation, mitochondrial metabolism, and apoptosis [10]. Expression of aquaporins was found to be altered in tumors of various organs such as brain [11], colon [12], breast, prostate, lung, and ovary. AQP1, AQP5 and AQP9 expression was found to be higher in malignant and borderline ovarian tumors compared to benign ovarian tumor and normal ovaries [13]. AQP1 was predominantly expressed in microvascular endothelium of the ovary tissue but found rarely in ovarian tumor cells [13,14]. AQP9, an aquaglyceroporin responsible for glycerol and water transport expressed in normal ovarian surface epithelium, was found to be overexpressed in borderline and malignant tumors [13]. Similar to AQP9, AQP5 expression was reported to be higher in ovarian tumors associated with lymph node metastasis [13].

Laying hens are emerging to be the most appropriate animal model for human ovarian cancer as they develop ovarian tumor spontaneously similar to women with a very high rate of incidence as high as 50% during 2–4 years of age [15-17]. Laying hens mimic human ovarian tumor, exhibiting similar disease progression and histological features [18]. Advanced stages of ovarian tumors in chickens as in human are typically associated with the presence of ascites. Recently, we have characterized various ovarian tumor cell lines obtained from ascites of laying hens that had advanced stages of ovarian tumors [19]. Large volumes of ascites, as high as 800 ml or approximately 70% of body weight, accumulate in the viscera of chickens at advanced stages of ovarian tumor [19]. Since greater AQP5 expression has been positively correlated with ascites volume and lymph node metastasis in human subjects with ovarian tumor [13], we sought to elucidate the expression of AQP5 in the chicken model of ovarian tumor and in ascites-derived chicken ovarian cancer (COVCAR) cell lines. We hypothesized that AQP5 expression is altered in COVCAR cells and in cancerous ovaries obtained from highly metastatic ovarian tumors compared to normal ovarian surface epithelial (NOSE) cells and normal ovaries, respectively. The objective of this study was to determine cellular localization of AQP5 in normal and cancerous chicken ovaries and to determine if AQP5 mRNA and protein quantities are different in chicken ovarian tumors and in COVCAR cells compared to that in normal ovaries and NOSE cells, respectively.

## Materials and methods

### Animals

Single-comb White Leghorn chickens were provided unrestricted access to feed and water at all times and maintained at 16 h light and 8 h dark cycle. All animal procedures were carried out in accordance with the Institutional Animal Care and Use Committee approved protocol.

### Tissue collection and cell culture

Normal and cancerous ovaries were collected from chickens (3–4 years-old; n = 5) as described previously [19]. After collection, normal and tumorous ovaries were either frozen in liquid nitrogen and stored at −80°C for extraction of protein and RNA or fixed in Bouin’s fixative solution for histopathology. Ovary tissue fixed in Bouin’s fixative solution was dehydrated and embedded in paraffin to prepare 4–6 μm thick tissue sections for histology and immunohistochemistry. Ascites was collected aseptically for isolation and culture of ovarian cancer (COVCAR) cells, as described previously [19]. COVCAR cells lines (C5, C6, C7, C11, and C19) were cultured in MCDB105-M199 cell culture medium at 37°C and 5% CO_2_. Upon reaching 80-90% confluence, the cells were passaged and replated at 1:1 or 1:2 ratio. Total RNA and protein were extracted from COVCAR cells (passages 3–4) as described previously [19]. To serve as control for COVCAR cells, normal ovarian surface epithelial (NOSE) cells were obtained by gentle scraping of the surface of pre-ovulatory follicles collected from regularly ovulating healthy chickens*.* The NOSE cells were cultured under identical conditions as used for COVCAR cell culture until they reached 80-90% confluence before preparation of cellular lysates for protein and RNA extraction. We have previously characterized the COVCAR cells lines (C5, C6, C7, C11, and C19) as to their invasiveness in extracellular matrix, ability to grow in soft agar and elevated expression of several ovarian tumor associated genes or proteins including E-cadherin [19].

### Histopathology and immunohistochemistry

Normal and cancerous ovary tissue sections (n = 5 animals) were deparaffinized in Histoclear (Electron Microscopy Sciences, Hatfield, PA) and hydrated using descending concentrations of ethyl alcohol in water. Tissue sections were stained with hematoxylin and eosin for histopathological examination by a board-certified veterinary pathologist (Dr. Timothy Cooper, Hershey Medical Center, Pennsylvania State University). For immunohistochemical staining, an antigen retrieval procedure was performed by boiling tissue sections in 10 mM sodium citrate solution (pH 6.0) in a pressure cooker. Endogenous peroxidase activity was quenched using 3% H_2_O_2_ in methanol. After several washes in Tris-buffered saline (TBS) containing 0.5% Triton X-100 (TBSX; Sigma-Aldrich), tissue sections were incubated in 1% normal horse serum in TBSX at ambient room temperature followed by overnight incubation in goat anti-human AQP5 antibody (2 μg/ml; cat # SC-9891, Santa Cruz Biotechnology, Santa Cruz, CA). The immunogen used for producing anti-human AQP5 antibody is 89% homologous to the respective chicken AQP5 protein sequence. After several washes, tissue sections were incubated in biotinylated anti-goat secondary antibody (6 μg/ml, Vector Laboratories, Burlingame, CA) in TBSX. Tissue sections were then incubated in avidin-peroxidase supplied in Vectastain ABC elite kit (Vector Laboratories) followed by treatment with 3,3’-diaminobenzidine substrate solution (Vector Laboratories). After dehydration of tissue sections, the slides were visualized and photographed in an Axioskop microscope (Zeiss, New York, NY). For negative control, ovary tissue sections were incubated in 1% normal horse serum (Vector Laboratories) in TBSX for 1 h at ambient room temperature followed by overnight incubation in 1% normal horse serum (Vector Laboratories) in TBSX in place of goat anti-human AQP5 antibody.

### Real-time quantitative PCR

Total RNA was extracted from ovary tissue (cancerous and normal, n = 5) and cell lines (COVCAR and NOSE cells, n = 5) and reverse transcribed as described previously [19]. Briefly, 50 ng of cDNA prepared from total RNA extracted from each ovary tissue and cell line was mixed with 1X PerfeCTa SYBR Green Fastmix (Quanta Biosciences, Gaithersburg, MD), and 300 nM forward and reverse primers to amplify chicken AQP5 cDNA (Fwd: GCTCTGCTGTACTTCTACATCCTTGT, Rev: ATTTCTTCCTCTCCTCTCTCTGTTCT) or chicken β-actin (Fwd: CTGGCACCTAGCACAATGAA; Rev: CTGCTTGCTGATCCACATCT). Reactions were carried out in 7500 Fast-Real Time PCR System (Life Technologies) with the following thermal cycle: 95°C for 20 sec followed by 35 cycles of 95°C for 3 sec, 55°C for 10 sec and 63°C for 30 sec. At the end of amplification, a melting curve analysis was performed to confirm the presence of a single amplification product. Samples from each ovary tissue and cell line were run in triplicate to obtain average C_T_ values for AQP5 mRNA and β-actin mRNA. The log-linear threshold values (C_T_) during the exponential phase of the PCR for target mRNA were normalized to β-actin mRNA. AQP5 mRNA quantity was expressed as a proportion to β-actin quantity following 2 ^–ΔΔC^_T_ method for converting log-linear C_T_ values to linear term [20] and analyzed.

### Western blotting analysis

Protein lysates of ovary tissue (cancerous and normal, n = 5) and cell lines (COVCAR and NOSE cells, n = 5) were prepared as described previously [19]. The lysates were denatured, heated for 10 min at 95°C, and separated on a 10% Bis-Tris polyacrylamide gel (Invitrogen) in 3-(N-morpholino)propanesulfonic acid (MOPS) running buffer. Proteins were transferred to Immun-Blot polyvinylidene difluoride (PVDF) membrane and first incubated in 5% non-fat dry milk and then with affinity-purified goat anti-human AQP5 antibody (0.4 μg/ml; Santa Cruz Biotechnology). After several washes, membranes were incubated in horseradish peroxidase labeled donkey anti-goat IgG (0.08 μg/ml; Santa Cruz Biotechnology) and treated with WesternSure PREMIUM Chemiluminescent Substrate (LI-COR Biosciences, Lincoln, NE). Chemiluminescent signals were detected using the C-Digit blot scanner (LI-COR Biosciences) and analyzed using Image Studio Lite software version 3.1 (LI-COR Biosciences). Membranes were reprobed with mouse anti-chicken α-tubulin antibody (0.25 μg/ml; Sigma-Aldrich) followed by incubation in horseradish peroxidase labeled goat anti-mouse IgG (0.08 μg/ml; Pierce) and scanned as described above. AQP5 protein quantity was expressed as a proportion of α-tubulin levels and compared between cancerous and normal ovaries or between COVCAR cell lines and NOSE cells. To determine the specificity of AQP5 immunostaining, the primary antibody was preadsorbed with 2 μg/ml of AQP5 blocking peptide (cat # SC-9891, Santa Cruz Biotechnology) and used in Western blot analysis.

### Statistical analyses

All data were analyzed using SigmaPlot version 12 (Systat Software Inc, San Jose, CA, USA). Levels of AQP5 mRNA or protein in cancerous ovaries and COVCAR cell lines were compared with that of normal ovaries and NOSE cell lines, respectively, using student’s *t*-test. A probability level of P < 0.05 was considered as statistically significant.

## Results

### Ovarian tumor gross morphology and histopathology

**Figure 1:**
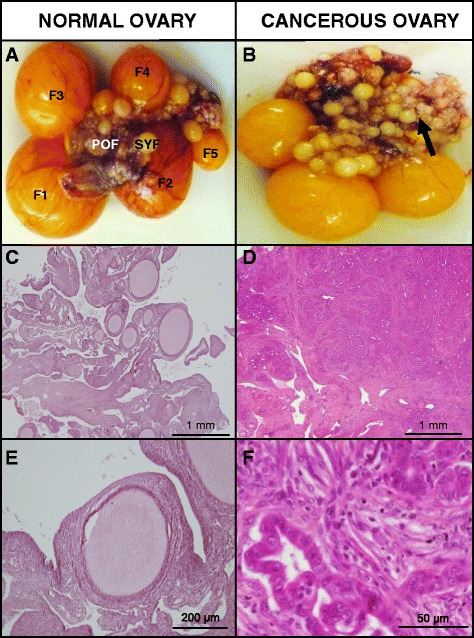
**Gross morphology and histology of normal and cancerous ovaries of chicken. A**. Normal ovary containing a hierarchy of preovulatory follicles (F1-F5), pre- hierarchical follicles (SYF), and post-ovulatory follicles (POF); **B**. Cancerous ovary containing tumor mass (arrow) in addition to a few atretic preovulatory follicles; **C-F**. Photomicrographs of normal **(C, E)** and cancerous **(D, F)** ovarian tissue sections stained with hematoxylin and eosin. SYF-small yellow follicle, POF-post-ovulatory follicle.

Representative photographs of normal ovary (Figure 1A) reveal a typical hierarchy of five preovulatory follicles and post-ovulatory follicular membrane whereas the cancerous ovary (Figure 1B) contained numerous prehierarchical follicles and cauliflower-like tumor mass. Photomicrographs of the normal ovarian stroma (Figure 1C and E) contained cross-sections of several ovarian follicles that have typical arrangement of theca and/or granulosa cell layers surrounding oocyte. In cancerous ovaries, the tissue was found to be expanded and nearly completely effaced by a large, invasive, unencapsulated, multinodular mass consisting of poorly differentiated epithelial cells arranged in tubules and acini separated by abundant stroma (Figure 1D). Epithelial cells appeared cuboidal with scant to moderate vacuolated eosinophilic to faintly basophilic cytoplasm and large round central nuclei (Figure 1F).

**Figure 2:**
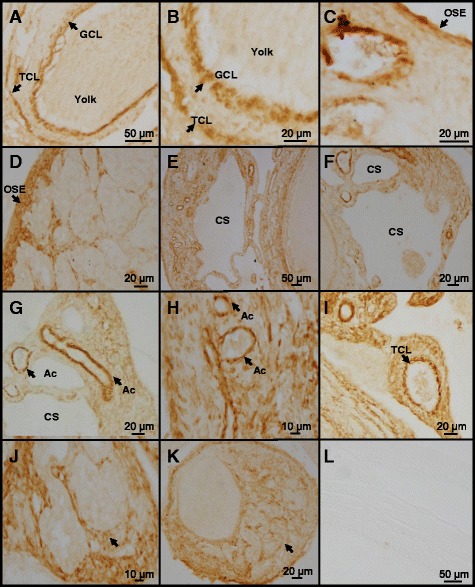
**Representative photomicrographs of normal and cancerous ovarian tissue sections showing AQP5-immunostained cells.** Bouin’s solution-fixed ovary tissue sections were immunostained using anti-human AQP5 antibody. AQP5 immunostaining (brown color) was visualized using a peroxidase-based detection system. **A-C**. Normal ovarian tissue sections exhibiting localization of AQP5 in granulosa and theca cell layers (arrows in **A** and **B**) and in ovarian surface epithelium **(C)**; **D-L**. Cancerous ovarian tissue sections exhibiting localization of AQP5 in ovarian surface epithelium (arrow in **D**), borders around cysts **(E, F)**, acini (arrow in **G** and **H**), theca cell layer (arrow in **I**), and in lobules of poorly differentiated neoplastic cells (arrows in **J** and **K**). Omitting anti-AQP5 antibody during the staining procedure abolished immunostaining completely **(L)**. GCL- granulosa cell layer, TCL-theca cell layer, OSE-ovarian surface epithelium, CS-cystic space, Ac-acinus.

### Localization of AQP5 immunoreactive cells in normal and cancerous ovaries

AQP5 immunoreactive (ir) cells were found predominantly in the granulosa and theca externa layers in normal ovarian prehierarchical follicles (Figure 2A and B). The pattern of AQP5 immunostaining appeared different in cancerous ovaries (Figure 2D-L). Several layers of AQP5-ir cells were found stacked under the OSE (Figure 2D) in contrast to a thin layer of AQP5-ir cells in the normal OSE (Figure 2C). Flattened cells lining the cystic spaces in ovarian tumor were found to be strongly immunoreactive to AQP5 (Figure 2E, 2 F). Several acini-like structures uniformly contained AQP5-ir cells (Figure 2G and H). Some of the prehierarchial follicles in cancerous ovaries displayed strong AQP5 immunoreactivity in the theca cell layer (Figure 2I) while others showed distended theca cell layer containing several AQP5-ir cells (Figure 2J). AQP5-ir cells were also found in flattened cells surrounding tumor nodules (Figure 2K). Omission of anti-AQP5 antibody completely abolished immunostaining (Figure 2L) confirming the specificity of anti-human AQP5 antibody in detecting chicken AQP5.

### AQP5 mRNA and protein expressions in cancerous ovaries

**Figure 3:**
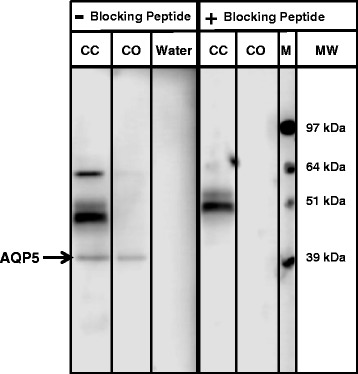
**Western blot analyses to detect AQP5 in cancerous ovaries and ascites-derived chicken ovarian cancer (COVCAR) cells.** Protein extracts from ovary tissue and COVCAR cells were subjected to Western blot analysis under reducing conditions. AQP5 protein was detected by immunostaining using anti-human AQP5 antibody (−Blocking Peptide) and chemiluminescence. Specificity of AQP5 antibody was determined by preadsorption of anti-human AQP5 antibody with human AQP5 peptide (+Blocking peptide). CC-COVCAR cell lysate, CO-cancerous ovary lysate, M- molecular weight.

**Figure 4:**
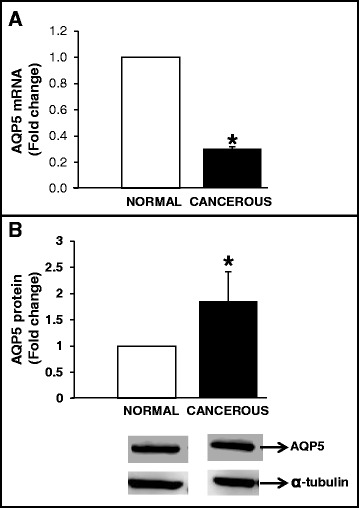
**Quantification of AQP5 mRNA and AQP5 protein in cancerous ovaries. A**. Quantification of AQP5 mRNA in cancerous ovaries. Total RNA extracted from cancerous and normal ovaries (n = 5 animals), was DNAse-I digested and reverse transcribed. Following reverse transcription, approximately 50 ng of cDNA was used in quantitative real-time PCR using SYBR® green as the dye to quantify AQP5 mRNA and β-actin mRNA in separate reactions. Each reaction was run in triplicate per tissue sample; and the critical threshold (*C*_T_) values, averaged, normalized to that of β-actin mRNA and converted from log-linear to linear term; **B**. Quantification of AQP5 protein in cancerous and normal ovaries. Protein extracts from cancerous ovaries (n = 5 animals) were subjected to Western blot analysis under reducing conditions. Chicken AQP5 protein was detected by immunostaining using anti-human AQP5 antibody and chemiluminescence. Alpha-tubulin was used as loading control. Data are represented as mean ± standard error of the mean from 5 replicates. *P < 0.05; cancerous versus normal ovary.

AQP5 protein in cancerous and normal ovaries appeared as 39 kDa band (Figure 3). Pre-adsorption of anti-human AQP5 antibody with human AQP5 peptide completely abolished the AQP5 immunostaining in ovarian lysate (Figure 3). AQP5 mRNA levels were significantly lesser in cancerous ovaries compared to that in normal ovaries (n = 5; Figure 4A). Interestingly, AQP5 protein levels were approximately 2-fold greater in cancerous ovaries compared to that in normal ovaries (Figure 4B).

### AQP5 mRNA and protein expressions in COVCAR cell lines

**Figure 5:**
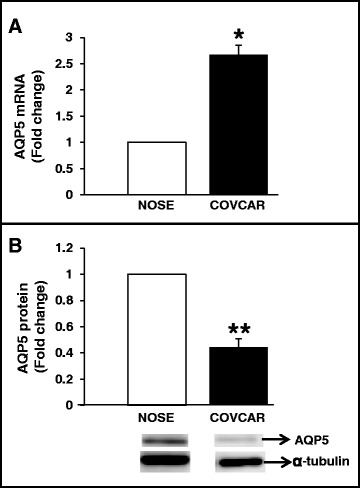
**Quantification of AQP5 mRNA and AQP5 protein in ascites-derived chicken ovarian cancer (COVCAR) cells. A**. Quantification of AQP5 mRNA in COVCAR cells. Total RNA extracted from COVCAR cells (n = 5 cell lines; C5, C6, C7, C11, and C19) and normal ovarian surface epithelial cell lines (NOSE; n = 5 cell lines), was DNAse-I digested and reverse transcribed. Following reverse transcription, approximately 50 ng of cDNA was used in quantitative real-time PCR using SYBR® green as the dye to quantify AQP5 mRNA and β-actin mRNA in separate reactions. Each reaction was run in triplicate per cell line; and the critical threshold (*C*_T_) values, averaged, normalized to that of β-actin mRNA and converted from log-linear to linear term; **B**. Quantification of AQP5 protein in COVCAR cells. Protein extracts from COVCAR cells (n = 5 cell lines; C5, C6, C7, C11 and C19), and NOSE cells (n = 5 cell lines) were subjected to Western blot analysis under reducing conditions. Chicken AQP5 protein was detected by immunostaining using anti-human AQP5 antibody and chemiluminescence. Alpha-tubulin was used as loading control. Data are represented as mean ± standard error of the mean from 5 replicates. *P < 0.05; **P < 0.001 COVCAR versus NOSE cells.

AQP5 mRNA concentration was found to be approximately 2.5-fold greater in COVCAR cell lines compared to that in NOSE cells (Figure 5A). AQP5 protein in COVCAR cell lysates appeared as 39 kDa band, similar to one observed in cancerous or normal ovaries (Figure 4). In addition, unlike ovary tissue lysate, COVCAR cell lysates exhibited three other bands of masses approximately 48, 51, and 60 kDa (Figure 3) that are possibly variants of AQP5 or non-specific immunostaining as pre-adsorption of anti-AQP5 antibody with AQP5 peptide completely abolished the 39 and 60 kDa AQP5 bands but not the 48 or 51 kDa bands (Figure 4). Upon quantification, the 39 kDa AQP5 protein levels in COVCAR cells were significantly lesser than that in NOSE cells (Figure 5B).

## Discussion

This is the first report to describe AQP5 expression in the chicken ovarian tumor model. In the normal ovary of chicken, AQP5 expression was found to be localized in the OSE, granulosa and theca cells. Similar to chickens, normal pig ovaries express AQP5 in granulosa and flattened follicular cells of primordial follicles [21]. The role of AQP5 in granulosa or thecal cell function and the factors affecting AQP5 expression remain to be elucidated. Based on the cell types where AQP5 is expressed in the ovary, it is plausible that AQP5 is involved in water transport in the developing follicle, yolk deposition, and in steroidogenesis. Coincidentally, progesterone treatment alone or in combination with estrogen was found to increase in AQP5 expression in rat uterine tissues demonstrated by situ hybridization histochemistry [22]. Using a similar approach, an increase in expression of AQP5 was reported in the glandular epithelium of progesterone-primed uteri following estrogen exposure in ovariectomized mice [23]. Collectively, AQP5 expression appears to be regulated by gonadal steroids in female reproductive tract.

We found that cancerous ovaries of the chicken exhibited several layers of AQP5-ir cells extending from the ovarian surface inwards. A board-certified histopathologist has determined the histotype of the cancerous ovaries used in this study as endometrioid type of ovarian adenocarcinoma [19]. In addition to the surface epithelium, AQP5 was expressed in the ovarian stroma, acini and in the cell lining the cysts. Presence of AQP5 in cells lining the cysts may suggest a potential role of AQP5 in formation of cystic fluid and secretions possibly required for the survival and growth of cancer cells. Similar to the chicken ovarian tumor, AQP5 localization has also been demonstrated in human ovarian tumor with localization of AQP5 protein in basolateral membrane of epithelial layer in benign tumor and plasma membranes of borderline and malignant tumors [13]. However, factors affecting AQP5 expression in cancerous ovaries are yet to be understood.

Our data suggest that AQP5 mRNA concentrations were 3-fold lesser in cancerous ovaries compared to that in normal ovaries. In contrast to our findings, AQP5 mRNA expression levels were found to be elevated in malignant ovarian tumors in women and such an increase was positively correlated to the ascites volume [24]. Such discordance may be due to species variation and may possibly indicate that regulation of AQP5 gene transcription and AQP5 mRNA turnover is different in chickens compared to humans. Although AQP5 mRNA concentrations were significantly lesser in cancerous ovaries in the chicken model, AQP5 protein concentrations were found to be significantly greater in the cancerous ovaries compared to that in normal ovaries. The negative correlation between AQP5 mRNA and protein exhibited in the ovary tissue examined in our studies can be attributed to several factors such as post-transcriptional modification, mRNA stability and processing. Consistent with our findings, higher AQP5 protein expression has been reported in borderline and malignant cancerous ovaries in women [13]. Data originating from several *in vitro* studies suggests that AQP5 is associated with increased proliferation, migration and decreased apoptosis in various tumor cell lines. For instance, lentivirus-mediated AQP5-shRNA transduction or hyperosmotic stress induced by sorbitol in MCF7 breast cancer cell line lead to attenuation of cell proliferation and migration [25,26]. Overexpression of AQP5 enhanced whereas, inhibition of AQP5 by acetazolamide significantly attenuated the proliferation and migration of gastric carcinoma cells [26]. Similarly, overexpression of AQP5 resulted in increased cell proliferation whereas small interfering RNA (siRNA) against AQP5 reduced cell proliferation rate in K562 and LAMA84 chronic myeloid leukemia cells [27]. Tumor necrosis factor-α, a pro-inflammatory cytokine that is elevated in tumors of various organs including ovaries [28] and breast [29], has been found to decrease AQP5 expression in acinar cells of human salivary gland via histone H4 acetylation [30]. However, treatment of CAOV3 ovarian cancer cell line with cisplatin decreases AQP5 protein expression and growth rate [31]. Similarly, epigallocatechin gallate treatment decreases AQP5 mRNA concentrations causing a decrease in proliferation of SKOV3 ovarian cancer cells [32]. Based on the foregoing, it is imperative to understand the direct role of AQP5 in ovarian tumor cell physiology.

We have previously reported that the ascites-derived COVCAR cell lines used in the present study are highly proliferative and possess greater invasiveness in Matrigel extracellular matrix compared to that of NOSE cells [19]. Considering the potential role of AQP5 in tumor cell proliferation and migration, we examined the AQP5 mRNA and protein expression levels in the COVCAR cell lines. Our data suggest that AQP5 mRNA in COVCAR cell lines were 2.5-fold greater than that in NOSE cells. Epigenetic changes such as CpG methylation in the promoter region of the AQP5 gene affects AQP5 mRNA expression in such a way that hypermethylation leads to repression of the gene expression and hypomethylation leads to increased gene expression [33]. Greater AQP5 mRNA in COVCAR cells in our studies may be due to hypomethylation of AQP5 gene. AQP5 protein levels, however, were found to be significantly lower in COVCAR cells compared to that in NOSE cells. Discordant mRNA and protein levels are not uncommon and could be attributed to several factors such as increased turnover of AQP5 protein leading to feedback upregulation of AQP5 gene expression. It is intriguing that AQP5 protein concentrations are downregulated in the COVCAR cell lines but upregulated in the cancerous ovaries. COVCAR cells are derived from ascites and as such these floating metastatic cells may have adapted to thrive in ascites by downregulating AQP5 protein levels and restricting water transport across the cell membrane. However, the tumor cells within the ovary would still require a higher level of AQP5 for proliferation and migration. It is not clear what factors are responsible to decrease AQP5 protein levels in the COVCAR cell lines.

In summary, our data have revealed a differential distribution of AQP5-ir cells in the ovarian tumor tissue compared with normal ovary suggestive of a potential role of AQP5 in proliferation and invasiveness of ovarian tumor cells. Similarly, AQP5 mRNA or protein concentrations in cancerous ovaries and COVCAR cells were found to be different from normal ovaries and NOSE cells, respectively. Collectively, our data suggest that altered expression of AQP5 may influence ovarian tumor cell proliferation, migration, and survival in ascites. Future studies should focus on exploring the pathways involved in altered AQP5 expression and its potential role in tumorigenesis in the chicken model of ovarian tumor.
